# Pagetoid Squamous Intraepithelial Neoplasia of the Vulva as a Mimicker of Vulvar Extramammary Paget Disease: Two Cases with Basal Layer Sparing

**DOI:** 10.1177/10668969221137527

**Published:** 2022-12-07

**Authors:** Jahg Wong, Annick Pina, Marie-Hélène Mayrand, Kurosh Rahimi

**Affiliations:** 1 Department of Pathology, 5622 Université de Montréal, Montreal, Quebec, Canada; 2 Department of Gynecologic Oncology, 5622 Université de Montréal, Montreal, Quebec, Canada; 3 Department of Obstetrics and Gynecology, Université de Montréal and CRCHUM, Montreal, Quebec, Canada

**Keywords:** vulva, HSIL, VIN, Paget disease, pagetoid, p16, basal

## Abstract

Human papillomavirus-associated vulvar intraepithelial neoplasia (high-grade squamous intraepithelial neoplasia [HSIL] or VIN of usual type) is a lesion characterized by atypia extending from the basal layer to the upper epidermis. There are only rare reports of vulvar intraepithelial morphology exhibiting a pagetoid pattern of intraepithelial dissemination. We herein report two cases of vulvar HSIL in which a pagetoid pattern of spread and a largely uninvolved basal layer represented a diagnostic pitfall for extramammary Paget disease. Nuclear atypia reminiscent of HSIL in addition to expression of p16, KRT5/6, and p40 were however in favor of pagetoid HSIL. Although there is morphological and immunohistochemical overlap between these two entities, an accurate diagnosis is important, since an erroneous diagnosis of vulvar extramammary Paget disease may lead to an extensive workup comprising radiological imaging, colonoscopy, and cystoscopy.

## Introduction

Human papillomavirus (HPV)-associated vulvar intraepithelial neoplasia (high-grade squamous intraepithelial neoplasia [HSIL] or VIN of usual type) is a precancerous lesion characterized by atypia extending from the basal layer to the upper epidermis. Since vulvar HSIL has several mimickers, p16 immunohistochemistry can be employed when HPV-independent lesions are part of the differential diagnosis.^1,2^ Criteria for positive p16 in vulvar HSIL are similar to cervical HSIL, in which diffuse and continuous staining involving the parabasal and basal layer is considered as “block positivity.”^3^ We report two cases of vulvar HSIL in which a pagetoid pattern sparing the basal layer represented a diagnostic pitfall for extramammary Paget disease (EMPD). We discuss the morphological features and immunohistochemical profiles between these two entities which are managed differently.

## Methods

Two cases of vulvar pagetoid HSIL were retrieved from our institution's in-house and consultation files. Three cases of non-pagetoid vulvar HSIL and EMPD diagnosed within the past year were also retrieved as controls. Cases of pagetoid HSIL, non-pagetoid HSIL, and EMPD were stained with a panel of immunohistochemical markers consisting of BerEP4, BRST-2 (GCDFP-15), mCEA, KRT5/6, KRT7, KRT20, EMA, GATA3, HER2, p16, p40, S100, SOX10, and 34βE12 (Table 1). The stains were not reperformed if they had already been conducted for routine diagnostic purposes.

**Table 1.** List of Antibodies, Dilutions and Clones for Immunohistochemical Stains.

Antibody	Dilution	Company	Clone
BER-EP4	1-100	Dako	BER-EP4
BRST-2 optiview	1-1000	Cell Marque	EP1582Y
CEA(M) optiview	1-200	Genetex	COL-1
KRT5/6	1-200	Dako	D5/16B4
KRT7	1-200	Dako	OV-TL 12/30
KRT20	1-50	Dako	KS20.8
EMA optiview	1-250	Leica	GP1.4
GATA3 optiview	1-200	Cell Marque	L50-823
HER24B5	None	Ventana	Rabbit M4B5
p16	None	Cintec	E6H4
p40	1-50	Bio Care	B28
S100	1-2000	Dako	POLY
SOX10 optiview	1-50	Cell Marque	EP268
34βE12	1-100	Dako	34βE12

Abbreviations: CEA(M), monoclonal carcinoembryonic antigen; EMA, epithelial membrane antigen; KRT, keratin; SOX10, SRY-related HMG-box 10.

## Patient 1

**Figure 1.**
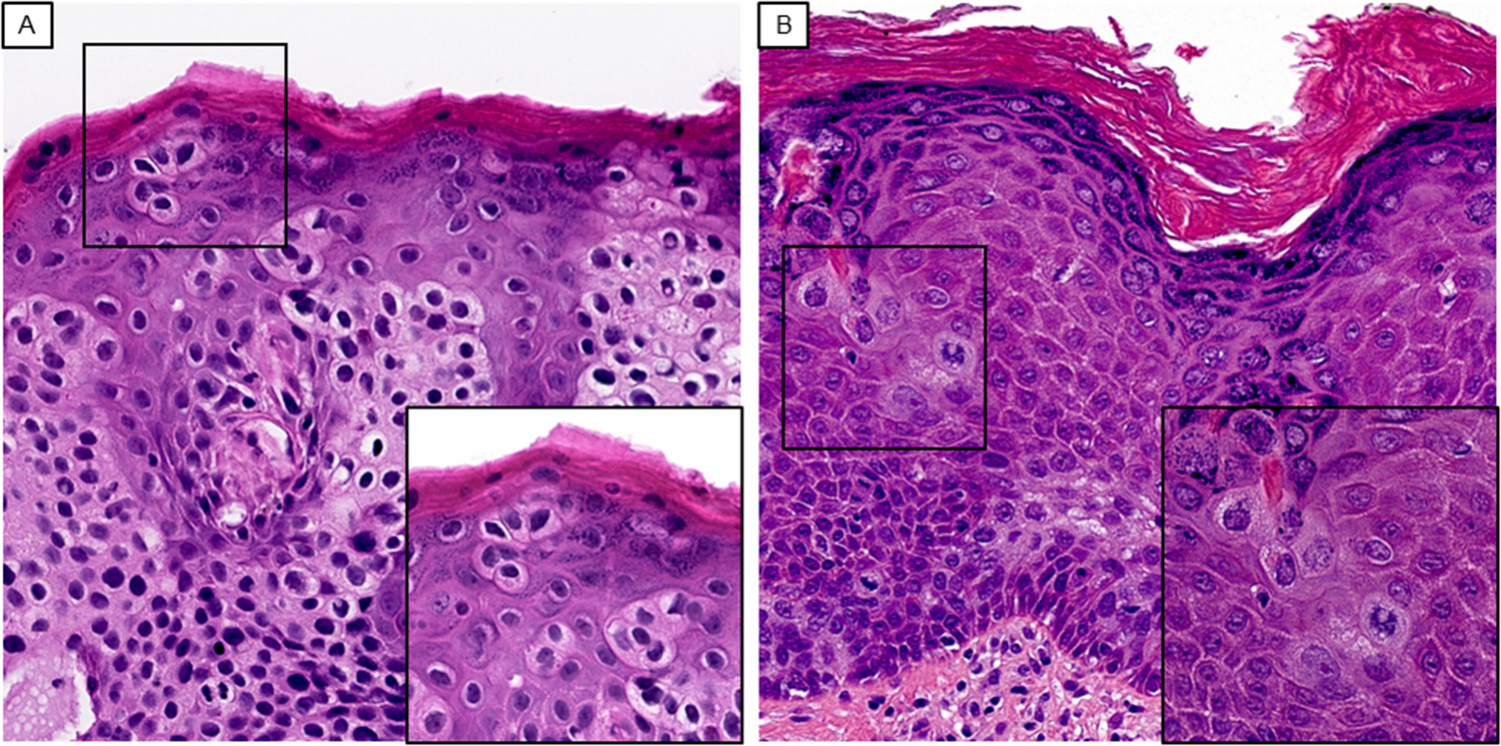
Pagetoid HSIL is characterized by nuclear atypia extending to the epidermal surface. The parabasal areas are composed of cells with hyperchromatic nuclei, scant cytoplasm, irregular nuclear contours, and marked pleomorphism without prominent nucleoli. Small clusters or individually scattered cells with more abundant cytoplasm bud off to infiltrate the epidermis in a pagetoid pattern (A and B from cases 1 and 2 respectively, H&E, original magnification x500, inlet magnification x800). Abbreviation: H&E, hematoxylin and eosin; HSIL, high-grade squamous intraepithelial neoplasia.

The first patient is a 77-year-old woman referred for further investigations after a biopsy of a benign appearing discolored patch of the vulva, performed in another institution, returned as uncertain but compatible with HSIL. Of note, the patient had a partial vulvectomy 3 years prior for non-pagetoid vulvar HSIL in our own institution. A first biopsy upon the patient's return to our institution showed melanosis. Because of increased pruritus, the patient consulted again at our center. The lesion had increased in size and showed changes compatible with vulvar HSIL. Histopathological examination of a second biopsy showed an epidermis populated with hypercellular zones alternating with normal ones. The cellular areas consisted of cells with overlapping hyperchromatic nuclei, irregular nuclear contours, and significant pleomorphism without prominent nucleoli. The atypical cellular zones occupied the parabasal layers, though extension to the epidermal surface was common. In addition, small cellular clusters with more abundant cytoplasm budded into superficial epidermis akin to a pagetoid pattern of spread (Figure 1). The basal and parabasal layers were largely uninvolved and the atypical cells extended into adnexal structures. Additionally, superficial mitotic figures were abundant and there were no foci of stromal invasion.

## Patient 2

The second patient is a 64-year-old woman who presented with vulvar pruritus and burning of 2 years duration. She had undergone biopsies on 5 different occasions over a 1-year period. The first three were indicative of HSIL, treated by excision then laser. The lesion returned after each treatment. The fourth biopsy was compatible with EMPD without invasive adenocarcinoma, but the fifth one indicated HSIL again. Of note, the patient was never more than a few months without a visible lesion. The patient was then referred to our unit, and was scheduled for colonoscopy, cystoscopy, and computed tomography imaging to determine if the Paget disease was primary or secondary. Furthermore, all previous biopsies and excision specimens were sent to our institution for slide review. Histopathological review of all specimens similarly showed hypercellular zones occupying the parabasal layers with extension to the epidermal surface. Similarly to our first patient, the cellular zones consisted of cells with overlapping hyperchromatic nuclei as well as irregular nuclear contours and inconspicuous nucleoli. These atypical cells showed scant cytoplasm though individual clusters of atypical cells budded into the most superficial layers of the epidermis in a pagetoid pattern. A monotonous basal layer was once again uninvolved by the hypercellular areas though adnexal structures were involved. There were numerous mitotic figures in the superficial epithelium and no stromal invasion was observed.

The histopathological findings of highly atypical cell clusters showing a pagetoid pattern of dissemination, raised suspicion for vulvar HSIL exhibiting a pagetoid pattern, vulvar EMPD, and vulvar melanoma.

**Figure 2.**
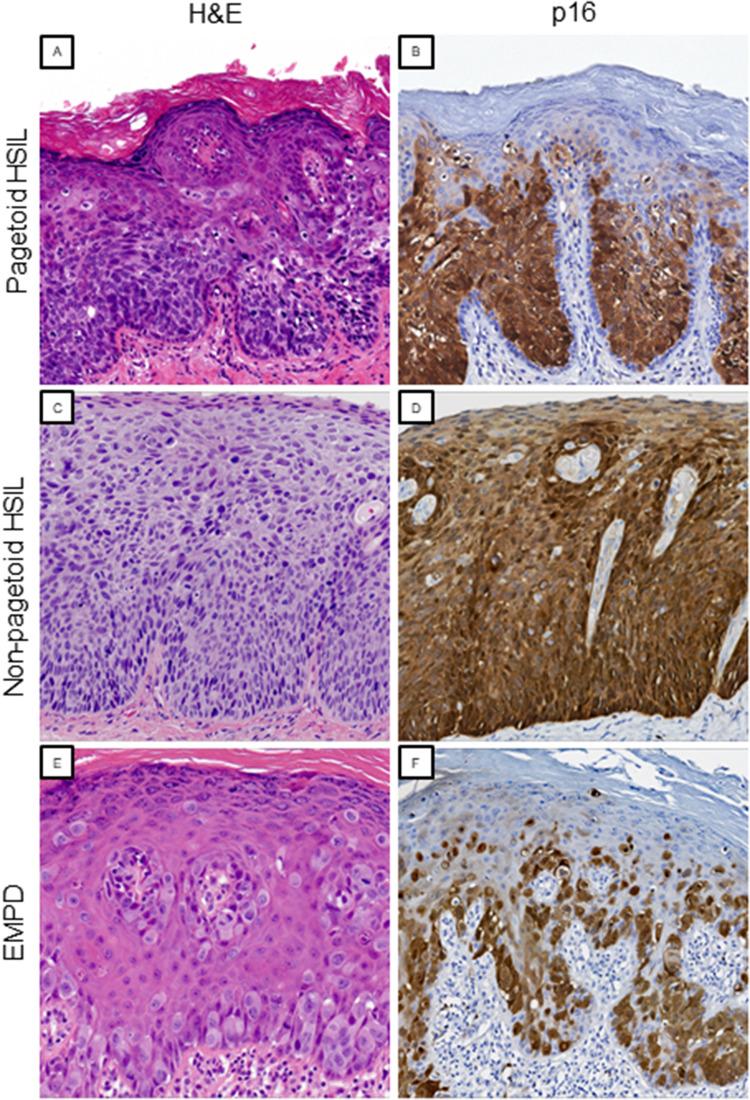
Expression of p16 is diffusely and continuously expressed in nuclei and cytoplasm of pagetoid HSIL with basal layer sparing (A and B, H&E and p16, original magnification x200). In contrast, non-pagetoid HSIL shows block positivity for p16 that involves the basal layer (C and D, H&E and p16, original magnification x200). Expression of p16 in EMPD is heterogenous and interpreted as negative (E and F, H&E and p16, original magnification x200 and x150, respectively). Abbreviations: EMPD, extramammary Paget disease; H&E, hematoxylin and eosin; HSIL, high-grade squamous intraepithelial neoplasia.

For both patients, the atypical cells showed a strong, diffuse, and continuous expression of p16 within nuclei and cytoplasm that extended to the most superficial aspects of the epidermis. The basal layer was however largely negative for p16 expression (Figure 2). The atypical cells were also positive for KRT5/6, 34βE12 (strong in case 1 and mildly in case 2), BerEP4, p40, and GATA3. KRT7 and EMA were only positive in cases 2 and 1, respectively. Furthermore, the atypical cells were negative for KRT20, GCDFP-15, mCEA, HER2, S100, and SOX10.

Despite the diffuse expression of p16, it was difficult to unequivocally differentiate pagetoid HSIL from EMPD due an unaffected and monotonous basal layer. However, presence of nuclear atypia reminiscent of vulvar HSIL as well as expression of KRT5/6 and p40 favored HSIL with pagetoid intraepithelial dissemination over vulvar EMPD. The final diagnosis in both cases was therefore “Pagetoid vulvar intraepithelial neoplasia.”

The first patient was treated with laser therapy, and a repeat biopsy 3 months later showed recurrence of pagetoid HSIL. These new lesions were again treated with laser therapy. The second patient underwent partial superficial vulvectomy which showed noninvasive pagetoid HSIL with positive margins and laser therapy. Follow-up data was not yet available for this patient.

### Morphological and Immunohistochemical Differences Between Pagetoid HSIL, Conventional HSIL, and Vulvar EMPD

We compared the morphological features and immunohistochemical profiles between pagetoid HSIL (n = −2), non-pagetoid HSIL (n = 3), and vulvar EMPD (n = 3) (Table 2). Pagetoid HSIL cells featured hyperchromatic nuclei with inconspicuous nucleoli whereas cases of EMPD exhibited vesicular chromatin with prominent nucleoli. In addition, cases of pagetoid HSIL had abundant mitotic figures and apoptotic bodies that were rare to absent in EMPD. Two out of three cases of EMPD showed intracytoplasmic mucin vacuoles which were not observed in pagetoid or non-pagetoid vulvar HSIL. Of note, the cells exhibiting pagetoid spread in pagetoid HSIL and EMPD showed less nuclear crowding, more abundant cytoplasm, and lower nuclear to cytoplasmic (N:C) ratios compared to conventional HSIL (Figure 2).

**Table 2.** Morphological and Immunohistochemical Features of Vulvar Pagetoid HSIL, Non-pagetoid HSIL, and EMPD.

	Pagetoid HSIL	Non-pagetoid HSIL	Vulvar EMPD
Morphological features			
Nuclear hyperchromasia	+ + +	+ + +	-
Prominent nucleoli	-	-	+ + +
Cytoplasmic vacuoles	-	-	+ +
Increased N:C ratio	-	+ + +	-
Nuclear crowding	+ +	+ + +	-
Mitotic figures	+ + +	+ + +	+
Apoptotic bodies	+ + +	+ + +	+
Immunohistochemical profile			
p16	2/2	3/3	0/3
KRT5/6	2/2	3/3	0/3
KRT7	1/2	0/3	3/3
KRT20	0/2	0/3	0/3
p40	2/2	3/3	0/3
GATA3	2/2	3/3	3/3
BerEP4	2/2	0/3	3/3
mCEA	0/2	0/3	3/3
EMA	1/2	3/3	3/3
BRST-2	0/2	0/3	2/3
HER2	0/2	0/3	3/3
S100	0/2	0/3	/3
SOX10	0/2	0/3	0/3
34βE12	2/2 (weak in case 2)	3/3	3/3 (weak)

Symbols: + + + , abundant, prominent;  + +, moderate; + , rare, isolated; - , absent.

Abbreviations: EMA, epithelial membrane antigen; EMPD, extramammary Paget disease; HSIL, high-grade squamous intraepithelial neoplasia; KRT, keratin; mCEA, monoclonal carcinoembryonic antigen; N:C ratio, nuclear to cytoplasmic ratio; SOX10 : SRY-related HMG-box 10.

Comparison of immunohistochemical profiles showed that cases of pagetoid and non-pagetoid HSIL, but not EMPD. The basal sparing pattern was only identified in pagetoid HSIL. In contrast to EMPD controls, both patterns of HSIL were positive for KRT5/6 and p40, while negative for mCEA, BRST-2, and HER2. Alternatively, cases of pagetoid HSIL and vulvar EMPD expressed KRT7 (only observed in 1 case of pagetoid HSIL) and BerEP4, which were all negative in cases of non-pagetoid HSIL.

## Discussion

Only three other cases of vulvar pagetoid HSIL have been documented in the literature. Raju et al^4^ reported the first case of pagetoid vulvar squamous cell carcinoma in situ, in which they noted that certain nests of atypical cells had an interposed layer of normal basal cells. The pattern of p16 expression was however not described. Two additional cases were reported by Armes et al,^5^ including one case associated with foci of invasive squamous cell carcinoma. In an analysis of 56 cases of anogenital HSIL, it was observed that up to 21% of anogenital HSIL may harbor a pagetoid scatter of atypical keratinocytes though it was not specified how many of these cases involved the vulva or whether these lesions were HPV associated.^6^ Pagetoid patterns of squamous intraepithelial neoplasia have been more frequently reported in sun-exposed skin without proclivity for HPV infection and may therefore reflect a different pathological process.^7^

p16 expression with a basal sparing pattern is not well described in vulvar HSIL. This pattern may however be observed in cutaneous squamous cell carcinoma in situ (SCCIS), or Bowen's disease, which is often associated with sun exposure.^8,9^ It is unclear whether p16 basal sparing in pagetoid HSIL reflects the activity of a high-risk HPV strain or other biological mechanisms that may be similar to those underlying sun-induced SCCIS.^5^

The distinction between pagetoid HSIL and EMPD may be difficult as there may be morphological and immunohistochemical overlap. For instance, a diffuse expression of p16 is conventionally indicative of HSIL but may be observed in EMPD.^10^ Conversely, KRT7 has been described to distinguish EMPD from vulvar squamous cell carcinoma, yet pagetoid HSIL may exhibit expression of KRT7 as seen in one of our own cases.^4,5,11^ Additional markers may therefore be useful in distinguishing pagetoid HSIL from EMPD. Previous studies have suggested that pagetoid HSIL can be favored with the expression KRT5/6, 34βE12, p63, and confirmation of HPV by polymerase chain reaction (PCR). Conversely, these same studies show that EMPD may express CAM5.2, CEA, GCDFP-15, HER2, and PASD.^4,5^ In our cases, KRT5/6 and p40 were diffusely positive in the atypical cells of pagetoid HSIL. 34βE12 was strongly positive in one case of pagetoid HSIL while the second case as well as all EMPD controls showed faint positivity. Furthermore, expression of GATA3 has been described to be common in EMPD though it is also expressed in non-pagetoid HSIL.^12^ Additionally, vulvar melanocytic lesions may sometimes mimic pagetoid HSIL and EMPD, though these lesions are positive for S100 and SOX10.

It is important to distinguish pagetoid HSIL from EMPD as they are managed differently. Therapeutic options for vulvar HSIL without suspicion of occult invasion include laser ablation, topical therapeutics, and excision.^13^ Although EMPD may be treated by excision or topical therapy, it is often associated with an underlying malignancy and may therefore require a potentially costly workup composed of radiological imaging, cystoscopy, and colonoscopy.^14^

The biological and prognostic implications in pagetoid HSIL are unknown due to a paucity of reported cases. The implicated HPV strain is known in only one reported case of pagetoid HSIL, which revealed the high-risk HPV type 73.^5^ Larger series are required to accurately assess the clinical implications of this rare morphological pattern.

In conclusion, we report two cases of pagetoid HSIL in which basal sparing on hematoxylin and eosin (H&E) sections and p16 immunohistochemistry simulated EMPD. In these cases, other markers such as KRT5/6 and p40 may further support a diagnosis of pagetoid HSIL. Failure to perform an extended immunohistochemical panel other than p16 may falsely lead to a diagnosis of EMPD and may lead to unnecessary investigations and treatments.
